# The relationship between electrophysiological and hemodynamic measures of neural activity varies across picture naming tasks: A multimodal magnetoencephalography-functional magnetic resonance imaging study

**DOI:** 10.3389/fnins.2022.1019572

**Published:** 2022-11-03

**Authors:** Tommi Mononen, Jan Kujala, Mia Liljeström, Eemeli Leppäaho, Samuel Kaski, Riitta Salmelin

**Affiliations:** ^1^Department of Neuroscience and Biomedical Engineering, Aalto University School of Science, Espoo, Finland; ^2^Aalto NeuroImaging, Aalto University, Espoo, Finland; ^3^Department of Computer Science, Aalto University, Espoo, Finland; ^4^Faculty of Biological and Environmental Sciences, University of Helsinki, Helsinki, Finland; ^5^Department of Psychology, University of Jyväskylä, Jyväskylä, Finland; ^6^BioMag Laboratory, Helsinki University Hospital, Helsinki, Finland

**Keywords:** multimodal data, data fusion, fMRI, MEG, picture naming, clustering, correlation patterns

## Abstract

Different neuroimaging methods can yield different views of task-dependent neural engagement. Studies examining the relationship between electromagnetic and hemodynamic measures have revealed correlated patterns across brain regions but the role of the applied stimulation or experimental tasks in these correlation patterns is still poorly understood. Here, we evaluated the across-tasks variability of MEG-fMRI relationship using data recorded during three distinct naming tasks (naming objects and actions from action images, and objects from object images), from the same set of participants. Our results demonstrate that the MEG-fMRI correlation pattern varies according to the performed task, and that this variability shows distinct spectral profiles across brain regions. Notably, analysis of the MEG data alone did not reveal modulations across the examined tasks in the time-frequency windows emerging from the MEG-fMRI correlation analysis. Our results suggest that the electromagnetic-hemodynamic correlation could serve as a more sensitive proxy for task-dependent neural engagement in cognitive tasks than isolated within-modality measures.

## Introduction

Functional magnetic resonance imaging (fMRI) and magnetoencephalography (MEG) are widely used non-invasive neuroimaging methods that both have their own strengths. FMRI measures hemodynamic modulations resulting from multiple neural and vascular phenomena, and yields an accurate three-dimensional map of brain activity with a good spatial resolution. However, its temporal precision is modest due to the sluggishness of the hemodynamic response. MEG measures the magnetic field elicited by electric activity, and possesses a temporal resolution in the millisecond scale. The distribution of cortical activity yielded by MEG is relatively smooth.

Thus, depending on the importance of the spatial and temporal aspects of brain activity to the research question at hand, either method may be the preferred option. It has also been proposed that combining MEG and fMRI would allow one to obtain accurate spatiotemporal maps of brain activity (Dale et al., 2000; Henson et al., 2010). Such approaches have, for example, utilized fMRI to spatially constrain the MEG source-level estimates, either explicitly (Matchin et al., 2019) or in a probabilistic manner (Cottereau et al., 2015; Wang and Holland, 2022). Another principle that has been applied to combine electrophysiological and hemodynamic signals is to use the MEG or electroencephalography (EEG) based individual measures of neural activity to model the fMRI response instead of common regressors across subjects, leading to increased statistical power (Renvall et al., 2012a; Iannaccone et al., 2015). In this effort, various kinds of computational analyses have been applied to obtain more comprehensive spatiotemporal accounts than can be afforded by MEG or fMRI data alone. In some instances, machine-learning based classification analyses have been conducted separately for MEG and fMRI data to obtain maximally accurate temporal and spatial accounts of neural phenomena (Brandman and Peelen, 2017). Computational models have been applied to identify electrophysiological correlates of behavioral processes which, in turn, have been used to model the trial-level variability within fMRI signals (Pisauro et al., 2017). Some studies have further utilized representational similarity analyses across MEG and fMRI data to accomplish spatially and temporally detailed characterization of neuronal activity (Cichy et al., 2014, 2016; Leonardelli and Fairhall, 2022). Generative models have also been utilized to capture the state transitions in both fMRI and MEG resting-state networks (Jiang et al., 2022). In a clinical setting, fusion of distinct neuroimaging measures, such as MEG and fMRI, is increasingly being used to improve the ability to distinguish between patient groups and control participants (Calhoun and Sui, 2016). Together, these reports demonstrate the versatile ways that MEG and fMRI can be merged to obtain spatiotemporally detailed accounts of neural-level processing.

In order to use electrophysiological and hemodynamic measures together in a principled manner, it is important to understand the relationship between the different types of measures. Numerous neuroimaging studies have therefore attempted to deepen this understanding. Initially, the emphasis was on how electrophysiological and hemodynamic techniques would allow the identification and localization of neural responses to the same types of stimuli (Sanders et al., 1996; Stippich et al., 1998). Subsequently, the focus has been more on identifying electrophysiological phenomena that correlate with the blood-oxygen-level dependent (BOLD) fMRI signal. In general, such studies have revealed robust and spectrally systematic correlation patterns between neural and hemodynamic activity (Logothetis et al., 2001; Mukamel et al., 2005; Scheeringa et al., 2011). However, it has also been shown that the correlation between electrophysiological measures and the BOLD signal varies across brain regions (Conner et al., 2011; Kujala et al., 2014). Moreover, comparisons between large-scale networks derived from MEG and fMRI have indicated a complex frequency-specific relationship between fMRI and the electrophysiological connectivity (Hipp and Siegel, 2015; Liljeström et al., 2015b). Furthermore, studies examining the MEG and fMRI signals in identical experimental settings from the same subjects have revealed systematic functional differences between the electrophysiological and BOLD responses (Liljeström et al., 2009; Vartiainen et al., 2011). Accordingly, it is commonly accepted that when integrating the temporally and spatially accurate views of neural processing from MEG and fMRI, it is crucial to consider the complex nature of the origins of hemodynamic fluctuations (Logothetis, 2008; Ekstrom, 2010; Lauritzen et al., 2012; Whitman et al., 2013).

One aspect that has received less attention in combining MEG and fMRI measures both for obtaining detailed spatiotemporal accounts as well as investigating neurovascular coupling has been the role of the applied stimulation or experimental tasks themselves. Naturally, a broad range of stimuli from different sensory modalities as well as various kinds of cognitive experiments have been applied. However, the main goal in those explorations has been to induce detectable signals in different neural systems across the cortex and to develop approaches that maximize the association between the two signal types (Lankinen et al., 2018), not to examine how the different stimuli and tasks might influence the joint modulation of electrophysiological and hemodynamic signals. Yet, it has been shown that the local neural and hemodynamic signals can be partially decoupled (O’Herron et al., 2016), and that the relationship between electrophysiological and hemodynamic signals depends on the correlation between the local inputs (Butler et al., 2017), effects that could cause variability in the MEG-fMRI correlations across stimuli and tasks. In the present study, we sought to explicitly utilize the inherently complex relationship between BOLD fluctuations and modulations of electrophysiological brain activity as well as the possible task-induced variability in this relationship to track and dissociate the neural engagement of different brain regions across distinct cognitive tasks. We asked whether any differences in neural processing related to distinct picture naming tasks could be highlighted through MEG-fMRI fusion as compared to isolated within-modality measures. Specifically, we investigated the variability of MEG-fMRI correlation patterns across three naming tasks (naming objects and actions from action images, and objects from object images) using a dataset where MEG and fMRI data were recorded from the same participants in identical experiments (Liljeström et al., 2009). The correlation patterns were obtained by first computing the MEG-fMRI correlation separately for each brain region, time window and frequency band, and by then applying variance minimizing hierarchical clustering to find clusters of similarly correlated brain areas. The approach allows the grouping of both task-invariant and task-dependent correlation patterns across brain regions regardless of their spatial adjacency. We predicted that our approach would reveal both types of correlation patterns and, critically, facilitate identification of neural engagement that could not be detected using one imaging modality alone.

## Materials and methods

### Subjects

Magnetoencephalography and fMRI data were collected from 10 healthy (nine right-handed, one ambidextrous), native Finnish-speaking subjects (four females, six males; ages 20–33 years). Informed consent was obtained from all subjects, in agreement with a prior approval of the Local Ethics Committee (Hospital District of Helsinki and Uusimaa). The subjects did not report any neurological disorders, and all had normal or corrected-to-normal vision. All methods were conducted in accordance with the guidelines of the Finnish National Board on Research Integrity.

### Experimental design

The task was to silently name pictures of objects or actions presented as simple black line art on a gray background. There were two categories of drawings. In the first category, an action performed with an object was depicted, whereas in the second category, a single object was shown. To achieve the same visual complexity as in action images the object images were constructed from the action images by dissolving the action figures into non-meaningful lines in the background. The experiment consisted of three different cognitive tasks: Object naming from object images (100 trials), action naming from action images (100 trials), and object naming from action images (100 trials). The experiment had a blocked design with 10 stimuli of the same task presented within each 30-s block. Each image was shown for 300 ms at 1.8–4.2 s intervals. Each block started with an instruction indicating the task for the block. The task blocks were separated by 21-s rest blocks. The experiment was divided into two runs, with different sets of stimuli in the two runs (150 images per run, 50 per task). The experimental design was identical in MEG and fMRI leading to two matched runs per subject. The order of the three naming conditions was randomized in both runs and silent naming was used to avoid muscular artifacts. A complete description of the experiment can be found in Liljeström et al. (2009). The design permits identification of effects that are related to the naming task (comparing action naming to both object naming conditions) and to the picture type (comparing object-only images to both action image conditions). Behaviorally, (overt) object naming from action images leads to longer reaction times than for naming objects from object images or actions from action images (Liljeström et al., 2015a), indicating that increased effort or additional processing is required when naming objects from action images compared to the two other tasks. It is therefore of interest also to compare the object naming from action image condition to the other tasks.

### Functional magnetic resonance imaging data collection

The MRI data were collected at the Advanced Magnetic Imaging Centre (Aalto University) with a Signa VH/i 3.0 T MRI scanner (GE Healthcare, Chalfont St Giles, UK). Anatomical MRIs were acquired using a T1-weighted 3D spoiled gradient-echo sequence. Functional MRI data were collected using a single-shot gradient-echo planar imaging sequence (TR 3 s, TE = 32 ms, FA = 90, slice thickness 3 mm, in-plane resolution either 3 mm × 3 mm, or 3.4 mm × 3.4 mm). The first five functional volumes were discarded from the analysis.

### Magnetoencephalography data collection

Magnetoencephalography data recordings were conducted using a 306-channel whole-head device (Elekta Oy, Helsinki, Finland) in a magnetically shielded room. The data were bandpass filtered to 0.03–200 Hz and sampled at 600 Hz. The temporal extension of the Signal Space Separation method (Taulu and Simola, 2006) was applied in order to suppress contributions from external artifacts. Eye movements were monitored with electro-oculogram (EOG).

### Functional magnetic resonance imaging and magnetoencephalography data analysis

**FIGURE 1:**
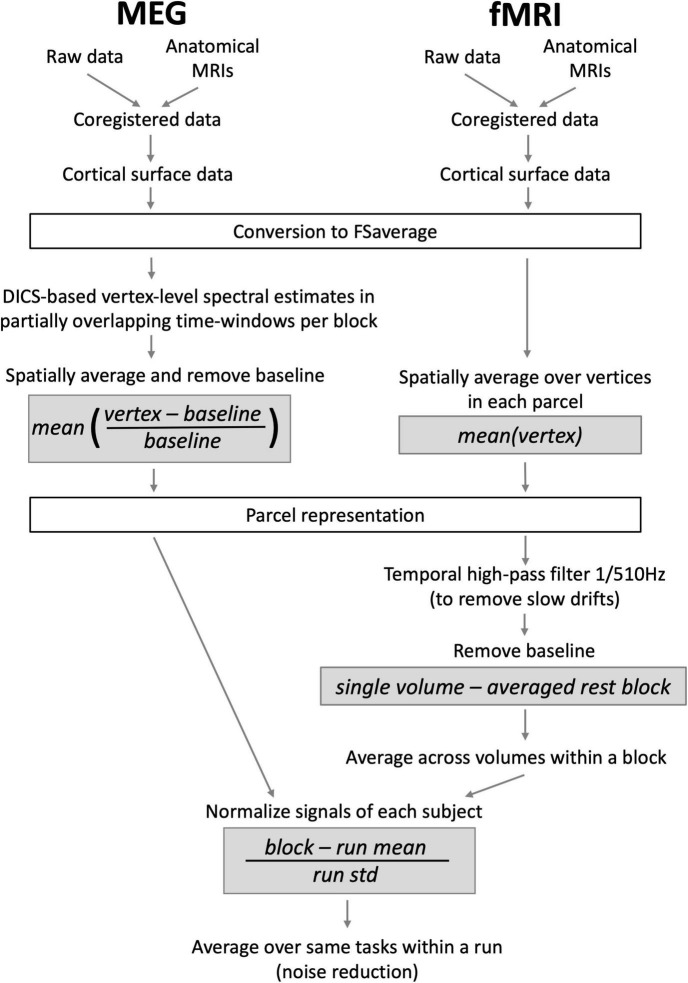
An outline of MEG and fMRI analysis pipelines, displaying the most important steps and their order. Gray boxes show essential normalizations that aim to equalize the measures obtained with the two modalities.

The overview of the analysis pipeline including key formulae for the conducted computations is presented in Figure 1. First, to facilitate the across-subjects evaluation of MEG-fMRI correlation, the data of each subject were transformed to an average brain *via* a surface-based transformation (Fischl et al., 1999) using Freesurfer 5.3 (Fischl, 2012). Before the transformation, the individual fMRI data were realigned to the first volume and susceptibility artifacts caused by movements were corrected for using SPM8 (Wellcome Department of Cognitive Neurology, London, UK). The mean image of the functional series was used for co-registering the fMRI data with the individual anatomical images. For each vertex in the average brain, the fMRI values of a spatially matching voxels were then taken to represent the fMRI activity at the cortical surface level.

The vertex-level data were averaged within 188 parcels covering the entire cortical surface (see e.g., Figure 3). This parcellation was based on the automatic anatomic parcellation of the human cortical gyri and sulci consisting of 144 parcels (Destrieux et al., 2010) that was subsequently computationally modified to form a parcellation that would be more suitable for the analysis of MEG data. Specifically, the parcellation scheme was obtained by applying a PCA algorithm implemented in MNE-python (Gramfort et al., 2013) for splitting parcels defined by the automatic anatomical labeling scheme of the cortical surface (Destrieux et al., 2010). The splitting yields parcels that are relatively symmetrical and small enough to be relatively homogeneous with respect to local activations. Notably, the splitting was based solely on the anatomical information without utilizing functional data, leading to a parcellation that is less optimized (Thirion et al., 2014) but more generalizable to multiple datasets. While the exclusively anatomical parcellation obtained *via* splitting the original parcels using spatial PCA does not ensure an exact alignment between MEG and fMRI responses and the parcels, it allows better separation of responses that are spatially distinct than when using the original Destrieux atlas. From the entire parcellation consisting of 188 parcels, regions that are prone to artifacts or signal loss in either of the imaging methods (anterior parts of the frontal lobe, deepest parts of the medial surface, and inferior parts of the temporal lobe) were omitted. The set of parcels used in the final analysis consisted of 70 parcels per hemisphere.

In the fMRI analysis the goal was to determine, for each fMRI run and experimental condition, the BOLD signal change with respect to rest within each parcel. This was accomplished by first high-pass filtering the parcel-level representation of the fMRI data in SPM8 with a cut-off frequency of 1/510 Hz. Baseline effects were removed using a rest block (6 volumes) that preceded each stimulus block (11 volumes), thus removing slow drifts taking place during the scanning runs. For each fMRI block and parcel, the data were averaged across the collected 11 volumes. The data were then normalized by subtracting the mean activity across all blocks and tasks from the block and task specific values, and by dividing these values by the standard deviation of the whole run’s data. The normalization was done separately for each subject to remove inter-subject differences in signal scales and means. Subsequently, within each run, blocks of each task were averaged per participant. For the correlation analysis, we thus obtained a total of 20 fMRI values per task (10 participants, 2 runs).

Magnetoencephalography data estimates were obtained for the same parcels in six different frequency bands: Theta (4–7 Hz), alpha (8–13 Hz), low beta (15–21 Hz), high beta (23–29 Hz), low gamma (36–46 Hz), and high gamma (54–90 Hz), from 100 to 800 ms with respect to stimulus presentation. The gamma-band analyses were conducted in two separate bands to avoid including 50 Hz line noise into the estimates. The estimation was done using event-related Dynamic Imaging of Coherent Sources (Laaksonen et al., 2008), a beamforming technique in the time-frequency domain. Here, only data from the 204 gradiometers were used. In the estimation, a surface-based grid consisting of 5,122 points was first created in the average brain with MNE (Gramfort et al., 2014) and transformed to each individual’s anatomy using Freesurfer 5.3 (Fischl, 2012). Brain activity estimates for each task and block were then computed for each grid point in the six different frequency bands, in 22 partially overlapping 200-ms time-windows (33-ms time difference between two successive time-windows). The 200-ms window length was chosen as it was the shortest length that allowed the accurate estimation of data covariance and thus brain activity given the signal-to-noise ratio (SNR) and number of trials across experimental tasks within the present dataset (see, e.g., Brookes et al., 2008). This window length is likely to be sufficiently short for exploring the sustained neural phenomena in higher-order cortical regions but could be sub-optimal for determining the temporally intricate early processes within the visual hierarchy. A baseline value was computed from the prestimulus interval −200 to 0 ms, separately for each block and grid point. Trials in which the amplitude of either the vertical or the horizontal EOG exceeded 150 μV were rejected. The parcel-level values were obtained by calculating, in each grid point, the difference between the post-stimulus values and the corresponding baseline values (divided by the same baseline values) and computing the average across all these baseline relative changes within each parcel. The parcel values were normalized separately for each subject, run and frequency band. This was done similarly as for the fMRI data, by subtracting the average activity across all blocks and tasks from the block and task specific values, and by dividing these values by the standard deviation of the data from the entire run. The run-level data were then obtained by averaging the block-specific data within each run. Similarly to the fMRI data, we thus obtained a total of 20 MEG values per task (10 participants, 2 runs) for the correlation analysis.

### Correlation analysis and clustering

**FIGURE 2:**
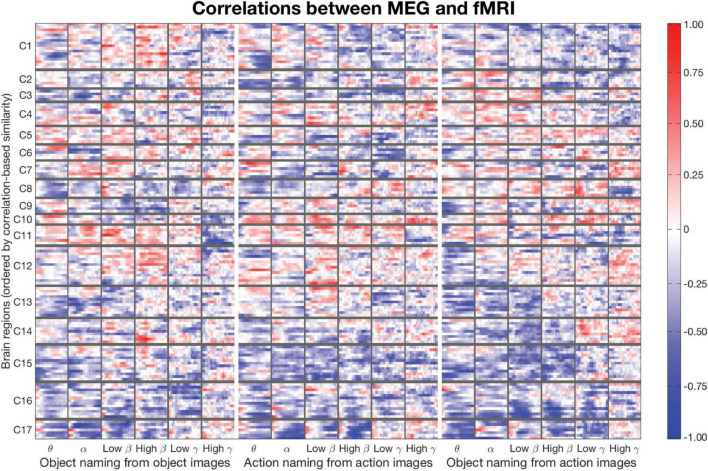
Matrix of correlations between MEG and fMRI for the three experimental conditions (separated with thick white vertical lines). Each condition-related submatrix is divided into six frequency bands: Theta, alpha, low beta, high beta, low gamma, and high gamma, from left to right (columns separated by thin vertical gray lines). Each frequency band consists of a sequence of 22 time points (sub-columns). All 140 brain regions (70 per hemisphere) are displayed on the *y*-axis, ordered with respect to the optimal leaf order of a cluster tree. This leads to a solution where distances between similarly behaving brain regions are minimized. The brain regions (rows) are divided into 17 clusters (C1–C17, separated by horizontal thin gray lines; see Figure 3 for visualization of the areas on MRI). The clustering is the same for all three conditions. The color indicates the MEG-fMRI correlation strength (–1…+1), see scale on the right.

We computed a vector of MEG-fMRI correlation estimates for each parcel using Spearman’s rank correlation (see Figure 2). Within a parcel, separate correlations were computed for all tasks, time intervals and frequency bands (3 tasks, 22 time-windows, 6 frequency bands: in total 396 MEG-fMRI correlation estimates per parcel). Each of these estimates was computed based on 20 MEG and 20 fMRI observations (10 subjects and 2 runs). We applied an agglomerative (merging) hierarchical clustering algorithm on our Spearman’s rho value vectors to find clusters of similarly correlated regions. For this, we used the Ward minimum variance method (Ward, 1963) that aims to minimize the within-cluster variance, leading to a clustering where correlation patterns inside a cluster are as similar as possible [function linkage(…,“ward”) in Matlab]. Information about hemispheres was not passed to the clustering algorithm. Ward’s method measures Euclidean distances between cluster centroids during its merging steps. The clustering algorithm produces a hierarchical cluster tree structure that describes the merging process. The leaves of the tree can be reordered without changing the structure itself. The optimal leaf order is such that the similarities of adjacent leaves are maximized [function optimalleaforder() in Matlab]. The final clustering allows for visual comparison of the correlation patterns across the three tasks and different frequency bands.

**FIGURE 3:**
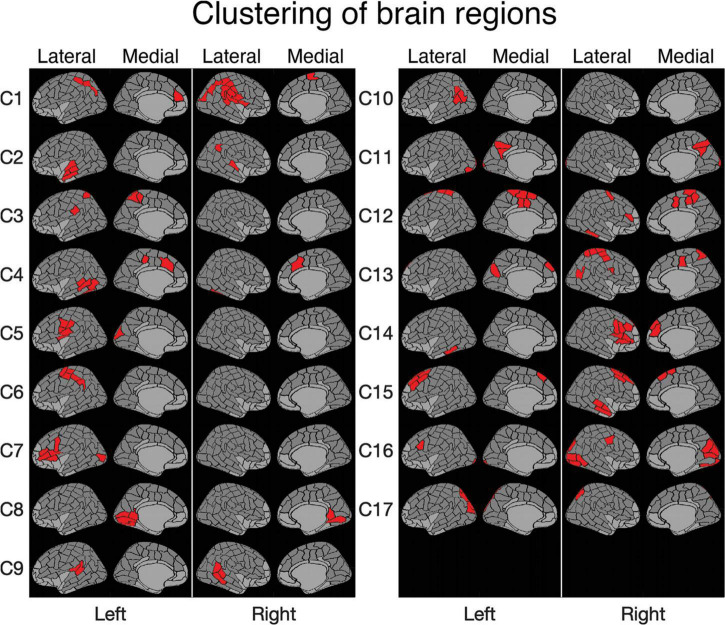
Clustering of brain regions. Level 17 of a clustering tree (used in the analyses). The deep medial and anterior frontal areas plotted in light gray were omitted from the analysis. Clusters are ordered according to the optimal leaf order and marked with labels C1–C17 (cf. Figure 2).

To evaluate the possible differences in correlations across the tasks, we estimated the 99% confidence limits for each task across the identified clusters, separately for left and right-hemisphere parcels, using bootstrapping (Efron, 1979). The bootstrapping was conducted by re-sampling the data 10,000 times, by computing the new MEG-fMRI correlation values for each sample, and by estimating the 99% confidence limits for each task from the obtained distribution. In the re-sampling, 80% of the data were randomly selected at each round. In this evaluation, we only considered those clusters and frequency bands in which at least one of the three tasks showed significant MEG-fMRI correlation (*p* < 0.05, Bonferroni-corrected over time points).

To compare a joint analysis approach and a more conventional approach utilizing a single brain imaging method alone, we also evaluated the differences in the MEG activity patterns between the tasks with paired *t*-tests (*p* < 0.05, Bonferroni-corrected over time points) for the identified clusters. This analysis was performed with the same temporal and spectral resolution as the MEG-fMRI correlation analysis and was, thus, only applicable to MEG; fMRI lacks the temporal resolution that would be needed for comparison of fMRI activity and MEG-fMRI correlation modulations. The comparison was therefore restricted to MEG activity and MEG-fMRI correlation patterns. Potential differences in the temporal-spectral aspects of the findings between the two approaches would reveal unique results that can be achieved only with one of the approaches, but not both.

## Results

### Clustering of correlation patterns

For clustering purposes, a matrix was constructed (see Figure 2), where each row lists the MEG-fMRI correlation values across the different frequency bands, time points and tasks. The clustering algorithm enables identification of clusters in which all three tasks behave similarly, but also clusters in which the tasks behave differently. In Figure 2, the rows are reordered according to a full cluster tree so that similar rows are close to each other. The ordering reveals salient MEG-fMRI correlation patterns, with consistent negative and positive correlation patterns across brain regions. The selected clustering consists of 17 clusters (Figure 3), chosen based on an appropriate level of spatial separation across parcels. With a smaller number of clusters, functionally distinct brain regions remain in larger shared clusters, whereas with a larger number of clusters single parcels start to form clusters by themselves. Accordingly, with a smaller set of clusters, regions with functionally distinct activity profiles would be merged together, whereas with a larger set of clusters individual parcels with very similar activity profiles would be segregated into distinct clusters. In general, the clusters were spatially concentrated, indicating that close-by regions show more similar MEG-fMRI correlation patterns than regions that are further apart. The clustering (Figure 3) agreed well with the known functional division of cortical processing related to picture naming, revealing, e.g., components representing both lower (C8) and higher-order (C16) visual, speech related motor/premotor (C5 and C7), and perisylvian language related processing (C9). In particular, lower-order regions involved in the basic visual processing formed clusters (C8, C11, C16, and C17) that did not include any higher-order cortical areas, whereas the clusters containing higher-order regions generally represented distinct neural functions associated with different cortical lobes and also with more fine-grained differences (e.g., separation of inferior vs. superior frontal cortices and lateral vs. medial cortical structures). Many of the identified clusters (C1, C4, C8, C9, C11, C12, C15, and C17) showed marked symmetry across the hemispheres, but temporal, central and inferior frontal cortical areas (e.g., C2, C5–C7, and C13–C14) critically involved in picture naming tended form clusters exclusively within individual hemispheres.

### Magnetoencephalography-functional magnetic resonance imaging correlation differences between tasks

**FIGURE 4:**
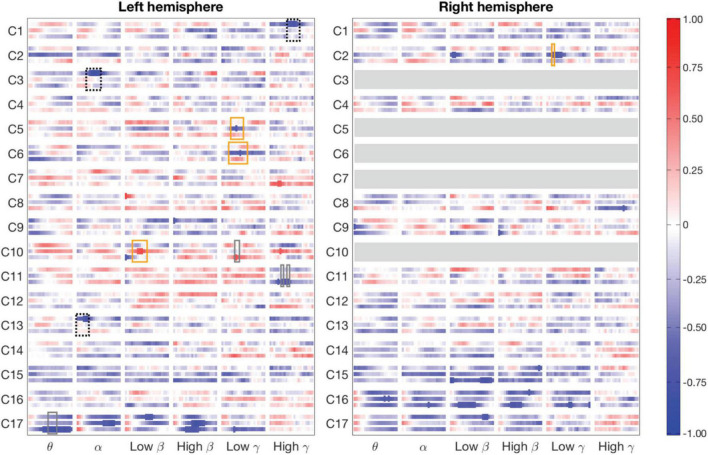
Magnetoencephalography-Functional magnetic resonance imaging correlation patterns divided into clusters (row labels) and hemispheres (left and right panels). The three rows in each cluster show correlation between fMRI and MEG for the three experimental conditions: from top to bottom, object naming from object images, action naming from action images and object naming from action images, over time in the different frequency bands (column labels). Significant correlations (*p* = 0.05, Bonferroni-corrected over the 22 time points) are marked as thicker parts of stripes. Rectangles indicate areas where the 99% confidence intervals of one condition do not overlap those of the other two conditions. A salient difference between naming tasks (naming actions vs. objects) is denoted by an orange rectangle, a difference between two picture types (action vs. object stimulus) is indicated by a dotted black rectangle, and a difference specific to naming objects from action images vs. the other two tasks with a gray rectangle. A rectangle is shown only when there is also a significant MEG–fMRI correlation inside the rectangle. Clusters C3, C5-C7, and C10 have parcels only in the left hemisphere (blank gray bars in the right-hemisphere).

For the clusters, we determined significant differences in MEG-fMRI correlation spectra between experimental conditions, across multiple frequency bands and time-windows (see Table 1 and Figure 4). We focused on identifying effects where one of the conditions differed from the other two conditions: (i) naming actions differed from both object naming conditions (different tasks; Figure 4, rectangles with solid orange line), (ii) naming objects from object pictures differed from naming objects or actions from action pictures (different images; Figure 4, rectangles with dotted black line), and (iii) naming objects from action pictures differed from both naming objects from object pictures and naming actions from action pictures (different reaction times; Figure 4, rectangles with solid gray line). Correlations were examined separately for parcels within each hemisphere.

**TABLE 1:** Significant effects detected with the given clusters, frequency bands, and significant time intervals.

Cluster	Hemisphere and primary location	Frequency band	Time (ms)	Confidence interval
**Correlation patterns specific to object images**				
C1	Left, superior parietal cortex	High gamma	300–600	99.9%
C3	Left, inferior parietal cortex and precuneus	Alpha	200–430	99.9%
C13	Left, cuneus and anterior frontal cortex	Alpha	100–370	99.9%
**Correlation patterns specific to action naming**				
C2	Right, superior temporal and inferior parietal cortex	Low gamma	100–300	99%
C5	Left, inferior precentral gyrus	Low gamma	230–430	99.9%
C6	Left, pre- and postcentral gyrus	Low gamma	300–500	99.9%
C10	Left, posterior temporo-parietal cortex	Low beta	200–470	99.9%
**Correlation patterns specific to naming objects from action images**				
C10	Left, posterior temporo-parietal cortex	Low gamma	230–430	99%
C11	Left, precuneus and occipital pole	High gamma	200–400	99%
		High gamma	300–500	99.9%
C17	Left, middle occipital cortex	Theta	330–570	99%

As significances are computed over 200-ms time-windows, it determines the lower bound for the size of significant time window. The correlation significance is Bonferroni corrected (*p* = 0.05) over 22 time-windows.

**FIGURE 5:**
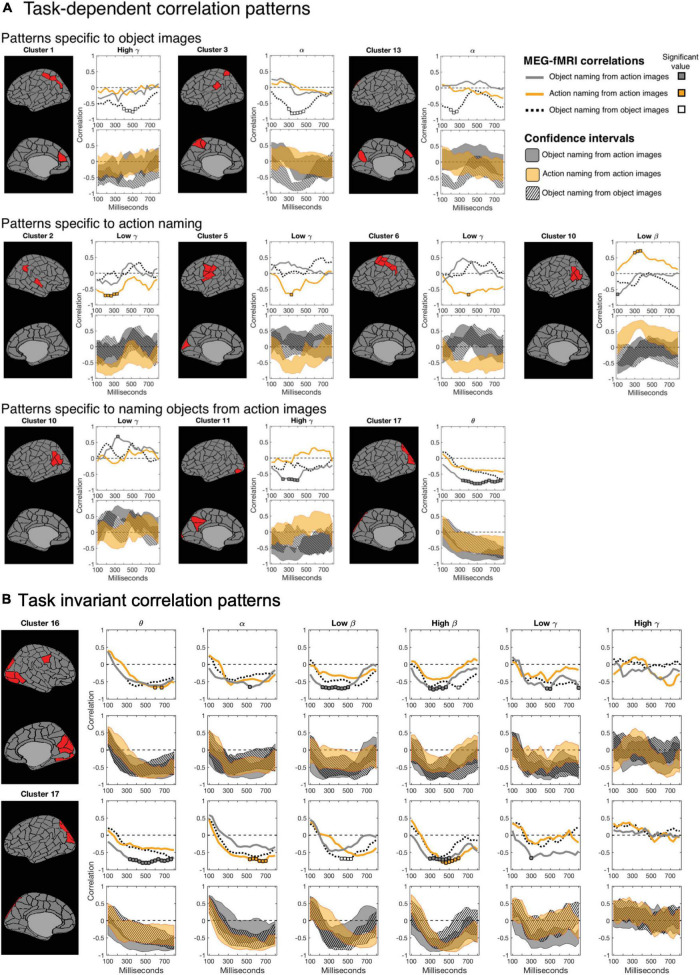
Magnetoencephalography-functional magnetic resonance imaging correlation as a function of time. For each cluster, the top row shows the correlation spectra for all tasks (naming object from action pictures in gray; naming actions in orange; and naming objects from object pictures in dotted black), and the bottom row the 99% confidence intervals for the three tasks (correspondingly gray, orange and a striped black pattern). In the correlation spectra, the colored squares indicate time instances at which the correlation is significant (*p* = 0.05, Bonferroni-corrected over time). **(A)** *Task-dependent instances*: One task shows significant correlation and differs from the other two tasks (non-overlapping confidence bounds) at a given time. White areas between the confidence intervals of experimental conditions indicate time instances of significantly different MEG-fMRI correlation between two or more conditions (*p* = 0.01, uncorrected). **(B)** *Task-invariant instances*: Clusters 16 and 17 suggest consistent negative correlation between MEG and fMRI at lower frequencies, among all experimental conditions, in the occipital cortex.

Modulations of MEG-fMRI correlation across-tasks were detected predominantly in the left hemisphere. Different picture types elicited distinct correlation patterns in the occipital and parietal cortex, within the alpha and gamma frequency bands (left-hemisphere clusters C1, C3, and C13; see Figure 5A and Table 1). Between different naming tasks, correlations differed along the central sulcus and the posterior temporo-parietal cortex, mainly in the left hemisphere (left-hemisphere clusters C5, C6, and C10 and right-hemisphere cluster C2), particularly in the gamma-range. Distinct correlation patterns for the condition in which the participants named objects from action images as compared to the other two categories were observed exclusively in the left hemisphere and included brain regions within the posterior temporo-parietal cortex (cluster C10) as well as within the occipital cortex (clusters C11 and C17), with contributions from the theta band as well as low and high gamma-bands.

### Task-invariant magnetoencephalography-functional magnetic resonance imaging correlation patterns

Figures 5B Shows the correlation patterns for two clusters within the occipital cortex (clusters C16 and C17). Parcels in the left-hemisphere cluster C17, covering the middle occipital cortex, and those in the right-hemisphere cluster C16, covering the medial and lateral parts of the occipital cortex, showed a significant negative MEG-fMRI correlation at low frequencies, but not in the gamma-range.

### Magnetoencephalography activation vs. magnetoencephalography-functional magnetic resonance imaging correlation

**FIGURE 6:**
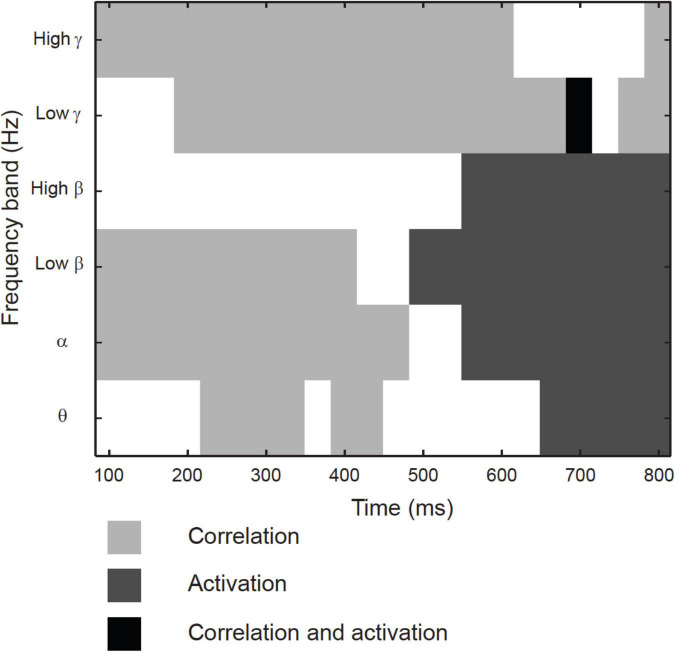
Temporo-spectral uniqueness and overlap in modulation of rhythmic activity and MEG-fMRI correlation. Timing with respect to picture presentation is plotted on the *x*-axis, and the different frequency bands on the y-axis. Time-frequency windows that showed differences between the conditions only for MEG band-limited power (light gray), only for MEG-fMRI correlation (dark gray) or both (black). Values averaged across all contrasts.

**FIGURE 7:**
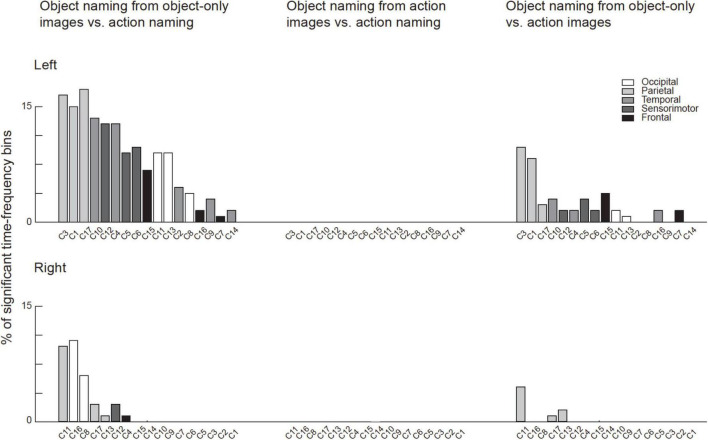
Significant results in the MEG activation analysis for each cluster. For each cluster the number of significant time-frequency bins are indicated as the percentage of all possible time-frequency bins (in total 132 bins from 6 frequency bands and 22 time-windows). The bars are color-coded according to the lobe to which the majority of the parcels belong to. The clusters are ordered according to the total percentage of significant time-frequency bins in all tasks (left and right- hemispheres separately). Note that there were no significant differences between neural activity during Object naming from action images and action naming, whereas for the other two contrasts where the stimulus contents were different multiple clusters showed significant differences.

Across the 17 identified clusters, the time-frequency windows in which MEG-fMRI correlation showed task-dependent modulation were highly distinct from the time-frequency windows in which MEG activity was modulated (Figure 6). Modulation of correlation was observed mainly in early time-windows (<500 ms), whereas modulations of activity (with band-limited power as measure) were exclusively detected more than 500 ms after stimulus onset. In the frequency domain, the MEG activity modulations were concentrated to the theta, alpha and (low and high) beta bands, whereas the MEG-fMRI correlation effects also showed a prominent contribution of gamma-band neural activity. No significant MEG-fMRI correlation effects were detected in the high beta band. Significant differences in activation were detected between object naming from object vs. action images, as well as for object naming from object images vs. action naming from action images; however, no differences were observed between object vs. action naming from action images (Figure 7). These effects were particularly prominent within the left hemisphere, predominantly in clusters with parcels in the parietal lobe. No significant effects of MEG signal changes were detected between object and action naming from identical images, in contrast to the MEG-fMRI correlation analysis which identified several left-hemisphere clusters in which action naming differed from the other two conditions (C2, C5, C10, and C13, Figure 5).

## Discussion

We have shown that correlation between MEG and fMRI contains information that distinguishes between the three naming tasks. This finding aligns with observations that have demonstrated trial and stimulus dependent variability in the relationship between electrophysiological and hemodynamic activity within the visual cortex (O’Herron et al., 2016; Butler et al., 2017). Furthermore, our results demonstrate that the time-frequency windows in which the MEG-fMRI correlation patterns differ between the tasks are distinct from the windows showing task effects in a separate MEG-based analysis of modulation of neural activity. Interestingly, the differences in the correlation patterns between tasks were typically observed in markedly transient time-windows, highlighting the dynamic nature of the neural phenomena dissociating the different picture naming conditions also at the level of MEG-fMRI correlations. Notably, such correlation differences were not specific to any frequency bands but extended to a wide range of distinct oscillations (theta, alpha, beta, and gamma). On the other hand, task-invariant correlations especially in the theta- and alpha-bands tended to be more sustained, attesting to the distinct nature of task-dependent vs. task-invariant correlation patterns. From amongst the 17 identified clusters, nine showed significant differences between the three experimental conditions whereas no differences were observed in the other clusters covering, in particular, more anterior lateral frontal areas and primary visual cortices. Significant differences were observed for all contrasts in the parietal cortex, with more superior effects for different images and more inferior effects for different tasks and conditions with different reaction times. Differences in the MEG-fMRI correlation patterns were also observed for different images in the anterior medial frontal cortex and for different tasks in the post- and precentral gyri. Notably, the involvement of the parietal cortex was detected also in the analyses focusing on the MEG and fMRI activity, whereas the role of the anterior medial frontal cortex and the post- and precentral gyri in dissociating the different naming conditions was not observed in these studies (Liljeström et al., 2008, 2009). Our results thus illustrate that the multimodal correlations yield novel information about the task-dependent neural engagement that cannot be detected using one imaging method alone.

### Detection of neural engagement using multiple neuroimaging methods

Task-dependent processing in neural circuits is a complex phenomenon that is supported by a wide range of mechanisms involving, e.g., electric, metabolic, and neurotransmitter activity (Singh, 2012). Measuring any of these processes yields one particular view of the full activity of the circuit. As it is not feasible to simultaneously record all possible processes related to the engagement of a circuit, its full activity remains a variable that may be estimated using specific proxies. As individual proxies are noisy and give incomplete information, it may not be possible to accurately estimate the full brain activity in a region. Thus, the observed activation patterns determined by an individual proxy may not reveal any observable brain activity even if the neural circuit, in reality, participates in task-dependent processing. The same holds when the goal is to determine differences between levels of neural engagement between different experimental conditions.

It has been proposed that the complexity of the human brain coupled with the incomplete measurements make multimodal data fusion critical for identifying detailed, individual-level properties of brain anatomy and function (Calhoun and Sui, 2016). Multimodal data-fusion based approaches have proven particularly useful for combining genetic mapping with other measures in the study of brain disorders (Purcell et al., 2009; Pearlson et al., 2015) as well as for evaluating the variability of brain anatomy and function in healthy subjects (Hardoon et al., 2009; Le Floch et al., 2012; Renvall et al., 2012b; Salmela et al., 2016) and predicting the subjects’ age (Engemann et al., 2020). So far, fusion of different neuroimaging data-types has been applied for identifying (in individual brain regions), e.g., the neural underpinnings of the BOLD response (Scheeringa et al., 2011; Kujala et al., 2014), also at the laminar level (Scheeringa et al., 2016; Warbrick, 2022), the effects of anatomical properties on functional data (Sepulcre et al., 2009; Schwarzkopf et al., 2012), or the effects of GABAergic inhibition on fMRI and MEG responses (Muthukumaraswamy et al., 2009; Kujala et al., 2015). While it has been proposed that by combining the temporally/spectrally and spatially sensitive measures of neural engagement provided by MEG and fMRI one could obtain a spatiotemporally accurate picture of brain activity (Dale et al., 2000), such data fusion has rarely been applied. Moreover, this type of combination has typically been used only in the primary sensory and motor neural systems (Schulz et al., 2004; Whittingstall et al., 2007; Stevenson et al., 2012; Renvall et al., 2012a; Cichy et al., 2014). Recently, similarity-based fusion methods combining MEG and fMRI have proven useful in learning relationships between visual objects and how they are represented within the visual system (Cichy et al., 2016) as well as within the semantic system (Leonardelli and Fairhall, 2022). In cognitive tasks, the improvement of SNR through group-level analysis (increased amount of data) may be limited by notable inter-subject variability, leading to a failure to detect the true engagement of neural circuits, even when multiple proxies are combined.

In the present study, we aimed to develop and apply a data-fusion based approach that would explicitly utilize the inter-subject and inter-block variability in combining different measurements (MEG and fMRI) to build a more sensitive and accurate picture of the neural engagement. Specifically, we used the correlation between MEG estimates of induced activity in different time-frequency windows and BOLD-fMRI estimates of hemodynamic activity to determine the neural circuits that are engaged in a distinct manner in three picture naming tasks. The MEG and fMRI proxies of neural activity can occasionally show salient negative or positive correlation when the brain activity is strong enough to be detected. In areas where one imaging method yields only noise and the other a good signal, task-wise correlations cannot be significant. To detect activity in a neural circuit, our approach requires that there is a causal connection between the engagement of the circuit and the two proxies (MEG and fMRI). Notably, unlike in typical neuroimaging studies, the sensitivity of the approach to detect neural engagement is in fact increased if the subjects or the blocks show considerable variability, given that the assumption of causality is met. In general, our approach as well as other approaches that profit from such variability are likely to be beneficial in cases where the SNR is low and where there is large individual variance in elicited neural processes. Hence, this type of approaches should prove useful in detecting neural engagement particularly in cognitive tasks.

### Multimodal correlation as a spatially, temporally and spectrally unique view on neural engagement during picture naming

In the present study, we applied the developed MEG-fMRI correlation based method to a picture naming data set that had been previously analyzed separately using traditional MEG (evoked responses) and fMRI group-level statistical approaches for identification of neural activity related to different naming tasks (action vs. object naming) (Liljeström et al., 2009) as well as identification of task-relevant functional networks (Liljeström et al., 2015a). Several studies have shown a negative correlation between MEG and fMRI at lower alpha and beta frequencies, and a positive correlation within the gamma frequency range, especially in low-level sensory cortices (Logothetis et al., 2001; Mukamel et al., 2005; Scheeringa et al., 2011). In higher-level cortical regions and in cognitive tasks this relationship is more variable (Conner et al., 2011; Kujala et al., 2014). Moreover, analysis of functional networks has indicated a complex frequency-dependent relationship between MEG- and fMRI-derived networks that varies across-tasks (Liljeström et al., 2015b). In the present study, we observed task-invariant negative correlations between MEG and fMRI within the alpha and beta frequency bands in occipital and parietal regions, in line with previous studies (Logothetis et al., 2001; Scheeringa et al., 2011).

Our main goal was, however, to utilize the variability in the relationship between MEG and fMRI and identify clusters that manifested a task-varying relationship in MEG and fMRI correlation. This correlation-based approach revealed significant differences between the conditions in which the activation based analysis had not done so. Within the left parieto-temporal junction, along the central sulcus, and the inferior frontal cortex, the correlation pattern was different between the action naming condition and the two object naming conditions. In contrast, MEG activation analysis either with induced responses in the present study, or previously with evoked responses (Liljeström et al., 2009), did not reveal significant differences between action and object naming from identical images. These effects demonstrate that the correlation-based analysis can reveal neural engagement in functionally relevant circuits that are not detected in conventional activation-based analyses.

The most notable new insights revealed by the present approach were the spectral and temporal patterns of electrophysiological activity. For example, the correlation patterns differed in the parieto-occipital cortex for the conditions where the stimulus content was different. In the present analysis of the modulation of induced activity, effects were detected in late time-windows (>500 ms), whereas the correlation patterns revealed differences primarily in notably earlier intervals (200–400 ms). These findings suggest that the modulation of alpha/beta activity is distinct for different stimulus contents, a finding that could not be inferred from traditional analysis of MEG activation; the results also demonstrate that these early differences are linked with the BOLD activity that is measured in those cortical regions. Secondly, the correlation-based analysis revealed, in contrast to analysis of MEG induced activity, prominent effects in the gamma-band. This suggest that the present multimodal analysis may help reveal the role of high-frequency neural activity in cognitive processing that is often difficult to detect with non-invasive techniques.

### Detection of cortical activity using clustering of magnetoencephalography-functional magnetic resonance imaging correlation patterns

In the present study, we computed the correlation between individual-level, run-wise MEG and fMRI recordings of the same experimental conditions from the same subjects. The goal was to develop an approach that would utilize the correlation between the two distinct proxies (MEG and fMRI) of brain activity to enhance the sensitivity of detecting the engagement of neural circuits in cognitive processing. The approach thus aims to capture effects related to the stimulus- and state-dependent input correlations and differences in the propagation of vascular dilation between neural columns (O’Herron et al., 2016; Butler et al., 2017) that would manifest as differences in the MEG-fMRI correlation patterns across experimental tasks. It should, however, be noted that our approach does not directly tell whether the circuit is more or less engaged during a task; the correlation-based measure can only reveal that the relationship between the applied proxies has changed. For example, our two proxies (MEG and fMRI) can be negatively or positively correlated, without indicating whether the amount of activity in the circuit has increased or decreased compared to the other conditions. In areas where one imaging method reveals only noise and the other detectable cortical activation, the task-wise correlations should not be significant. Our clustering approach corresponds to a conditioning which enforces the method to consider only those correlations that are related to the performed cognitive tasks. In the optimal situation, both proxies would have similar temporal granularity but, due to the highly integrative nature of the fMRI signal, precise temporal information was present only in the MEG signals. Nonetheless, we can track and utilize the temporal information in the MEG signals to dissociate even subtle effects in the integrative fMRI signals and, thereby, discover also small differences between cognitive tasks.

Spatially, our clustering-based analysis was designed to identify robust, large-scale effects in the correlation patterns that were specific to the given three naming tasks. Thus, the clustering results may not necessarily obey conventional knowledge about the locations of task-relevant functional brain regions. The reason is that the clustering is constructed using a very limited set of tasks. If these tasks do not distinguish between certain brain regions, then those regions will fall into the same cluster. Moreover, if the spatial extent of a cluster is too large, even a relatively strong signal may be masked by other contradicting signals or noise originating from the same cluster. If a cluster is too small, weak but significant signals may disappear as the region of activation has been split into parts. Some of the clusters are necessarily non-informative because none of the brain regions—including inactive regions—are left out in a clustering process.

In our study, we let the method cluster both hemispheres together. Thus, it is also possible that in some cases weaker, interesting signals might have been masked by stronger signals from the other hemisphere. Such a scenario could be avoided by conducting separate clustering for each hemisphere; however, this might hide some of the inter-hemispheric effects that were detected with the present approach.

## Conclusion

We introduced a correlation-based data-fusion analysis pipeline that utilizes two proxies of brain activity to enhance sensitivity for detecting the engagement of neural circuits in cognitive processing. Our results demonstrate that the approach discovers spatially, spectrally, and temporally unique task-specific information on cortical processing during picture naming. Multimodal data fusion based on correlations between electromagnetic and hemodynamic activity can thus reveal task-dependent neural engagement that may not be detected using the proxies of brain activity offered by one imaging method alone.

## Data availability statement

The data analyzed in this study is subject to the following licenses/restrictions: The MEG and fMRI data cannot be made openly available, according to the ethical permission and national privacy regulations at the time of the study, but are available from the corresponding author on reasonable request and with permission of the Ethics Committee of the Hospital district of Helsinki and Uusimaa. Requests to access these datasets should be directed to TM, tommi.mononen@helsinki.fi.

## Ethics statement

The studies involving human participants were reviewed and approved by the Ethics Committee of the Hospital district of Helsinki and Uusimaa. The patients/participants provided their written informed consent to participate in this study.

## Author contributions

TM, JK, ML, EL, SK, and RS: conceptualization and writing—review and editing. TM, JK, ML, EL, and SK: methodology. TM, JK, and ML: validation and formal analysis. ML: investigation. TM, JK, ML, and RS: writing—original draft. SK and RS: supervision and funding acquisition. All authors contributions is based on the CRediT taxonomy.
